# TECHNOLOGY AS A CATALYST FOR SUPPORTING AGING WITH DISABILITY

**DOI:** 10.1093/geroni/igad104.0125

**Published:** 2023-12-21

**Authors:** Tracy Mitzner

## Abstract

Individuals aging with long-term disabilities may need support to perform and participate in everyday activities associated with well-being and quality of life. Technology solutions have potential to effectively promote autonomy and aging-in-place for this population. This symposium will highlight technology research and development efforts from the Rehabilitation Engineering Research Center on Technologies to Support Aging-in-Place for People with Long-Term Disabilities (RERC TechSAge). TechSAge is dedicated to understanding the needs of, and developing supportive technologies for people aging with long-term vision, hearing, and mobility disabilities. Mumma et al. will present insights from interviews of adults aging with vision loss due to glaucoma and macular degeneration regarding their challenges and response strategies for everyday activities. Lee et al. will discuss findings from interview data exploring how wisdom is manifested from the lived experiences of individuals aging with disability (i.e., hearing, vision, and mobility). Mitzner et al. will describe characteristics of the participant sample from a clinical trial examining the effect of a Tele Tai Chi intervention on physical activity and social connectedness for adults aging with long-term mobility disabilities. Sanford et al. will present the findings from three TechSAge pilot projects focused on the use of technology to support in-home rehabilitation for individuals aging with long-term upper extremity disabilities: DigiHand, KeyStroke, and 3DP for AT. Together these studies illustrate the needs and coping strategies of older adults with disabilities and the potential of technology to support those needs. This is a collaborative symposium between the Lifelong Disabilities and Technology and Aging Interest Groups.

